# Targeting psychologic stress signaling pathways in Alzheimer’s disease

**DOI:** 10.1186/s13024-017-0190-z

**Published:** 2017-06-21

**Authors:** Hunter S. Futch, Cara L. Croft, Van Q. Truong, Eric G. Krause, Todd E. Golde

**Affiliations:** 1 Department of Neuroscience, University of Florida, Gainesville, FL 32610 USA; 2 Center for Translational Research in Neurodegenerative Disease, University of Florida, Gainesville, FL 32610 USA; 3 Department of Pharmacodynamics, College of Pharmacy, University of Florida, Gainesville, USA; 4 McKnight Brain Institute, University of Florida, 1149 Newell Drive, PO Box 1000015, Gainesville, FL 32610 USA

**Keywords:** Alzheimer’s disease, Corticotropin-releasing hormone, Psychologic distress, Chronic stress, Neurodegeneration

## Abstract

Alzheimer’s Disease (AD) is the most prevalent progressive neurodegenerative disease; to date, no AD therapy has proven effective in delaying or preventing the disease course. In the search for novel therapeutic targets in AD, it has been shown that increased chronic psychologic stress is associated with AD risk. Subsequently, biologic pathways underlying psychologic stress have been identified and shown to be able to exacerbate AD relevant pathologies. In this review, we summarize the literature relevant to the association between psychologic stress and AD, focusing on studies investigating the effects of stress paradigms on transgenic mouse models of Amyloid-β (Aβ) and tau pathologies. In recent years, a substantial amount of research has been done investigating a key stress-response mediator, corticotropin-releasing hormone (CRH), and its interactions with AD relevant processes. We highlight attempts to target the CRH signaling pathway as a therapeutic intervention in these transgenic mouse models and discuss how targeting this pathway is a promising avenue for further investigation.

## Background

Alzheimer’s Disease (AD) is a progressive neurodegenerative process that is currently the sixth leading cause of death in the United States [1]. The AD brain is characterized by the aggregation of misfolded proteins and marked neuronal loss [2]. The hallmark pathologic features of AD, intracellular neurofibrillary tau tangles (NFT’s), extracellular Amyloid-β (Aβ) plaques, synaptic and neuronal loss and reactive gliosis, have all been extensively studied. Despite this enormous scientific effort, many aspects of the disease remain poorly understood [3]. Although genetic factors clearly play a role in AD, studies of monozygotic twins have suggested that non-genetic risk factors are also involved [4, 5]. As non-genetic risk factors may be more easily modifiable than genetic risk factors, it is worthwhile to explore these pathways in search of therapeutic strategies for the treatment or prevention of AD. One non-genetic risk factor that has been associated with increased risk for AD is increased chronic psychologic stress. Epidemiologic studies have shown that individuals predisposed to experiencing psychologic stress, and those affected with diseases associated with chronic stress such as Major Depression and Post-traumatic Stress Disorder (PTSD), have an increased risk of developing AD (Table 1) [6–12].

**Table 1:** Relevant epidemiologic studies associating stress with increased risk of developing AD

Epidemiologic studies	Description	Results	Comments
Andel et al. (2012) [11]	Prospective study. Occupation used as a surrogate for stress level	Correlation between markers of Job Stress (Low job control, High demands, Low social support, High job strain) in risk of developing any type of dementia (OR 1.06–1.23) or AD (OR 1.04–1.23)	Study used only occupation of participant as surrogate for stress but did not survey participants directly about their stress levels. Clinical Diagnosis (Dx) of AD
Kaup et al. (2016) [7]	Prospective study. Elderly participants (Mean age 74) were assessed for depressive symptoms yearly for 5 years and then observed for an additional 11 years for onset of dementia	Patients found to have High and increasing symptoms were significantly more likely to develop any type of dementia (OR 1.94)	Clinical Dx of AD
Tsolaki et al. (2010) [12]	Retrospective Case–control study of patients with dementia and without.	Found that 78% of those with dementia had a stressful life event prior to onset of dementia while only 55% of the control subjects encountered a stressful life event. AD (OR 2.24)	Clinical Dx of AD
Wilson et al. (2003) [8]	Prospective study as part of the larger Religious Orders study of catholic nuns and priests, measuring stress proneness and incident AD.	Participants receiving the highest scores in the neuroticism scale used for a marker of stress proneness were twice as likely to develop AD within the 4.9 year average follow-up.	
Johansson et al. (2014) [9]	Prospective study examining stress levels of women at three examinations over 35 years.	Hazard ratio for incident dementia were 1.1 (.71–1.71) reporting stress at 1/3 examinations, 1.73 (1.01–2.95) at 2/3 examinations, and 2.51 (1.33–4.77) at 3/3 examinations.	Clinical Dx of AD

Although these studies associating psychologic stress and AD are intriguing, like most epidemiologic studies of AD these studies are subject to many confounds that could dampen enthusiasm for further research let alone for guiding a therapeutic development program. Indeed, unless based on autopsy proven AD or other modalities such as amyloid imaging that are now available to enable more accurate diagnosis of AD in living patients, most epidemiologic studies likely included a relatively high percentage of individuals (20% or more) with other forms of dementia and even the control group (>20%), if over age 65, is likely to have amyloid pathology [13, 14]. Thus, these studies are conservatively viewed as epidemiologic associations with dementia and not necessarily AD. Further, epidemiologic studies of psychologic stress are particularly challenging, as it is difficult to quantify stress in a way that is potentially reflective of the biological processes associated with this relatively subjective phenotype. Finally, in a disease like AD with a long prodromal state, it can be challenging to link early or mid-life risk factors with development of the disease late in life.

Given these and other potential confounds, epidemiologic data likely needs to be combined with biological or genetic data to serve as a basis for developing novel AD therapies. In this review we will evaluate the biological underpinnings that suggest the association between AD and stress may be strong enough to warrant investigation as a possible avenue for AD therapy. The course of molecular research into stress and AD began largely with investigation into stress-steroids, namely cortisol, but more recently has shifted towards Corticotropin-Releasing Hormone (CRH), a neuropeptide which exerts its effects primarily through activation of the CRHR1 receptor [15]. These data have shown that stress mediators are able to regulate AD relevant processes and provide converging evidence that activation of the stress response pathway may be able to exacerbate AD pathologies.

## Main text

### Stress signaling

When approaching the biology underlying the stress response, it is important to consider all of the potential contributors if we are to determine what pathways mediate the effects of stress on AD relevant processes. CRH and the CRH family peptides urocortin (UCN) 1, 2, and 3 are the main coordinators of the response to psychologic stress. These peptides exert their effects through binding and activating the CRHR1 and CRHR2 receptors in the central nervous system (CNS) and the periphery [15–17]. The classical stress response pathway begins with the perception of stress leading to the release of CRH from the paraventricular nuclei (PVN) of the hypothalamus, binding to CRHR1 Receptors in the anterior pituitary and causing the release of Adrenocorticotropic Hormone (ACTH) into the systemic circulation. ACTH then binds to MC_2_ receptors in the adrenal cortices, inducing glucocorticoid release [18]. This cascade is known as the Hypothalamic-Pituitary-Adrenal (HPA) axis [18, 19].

**Fig. 1:**
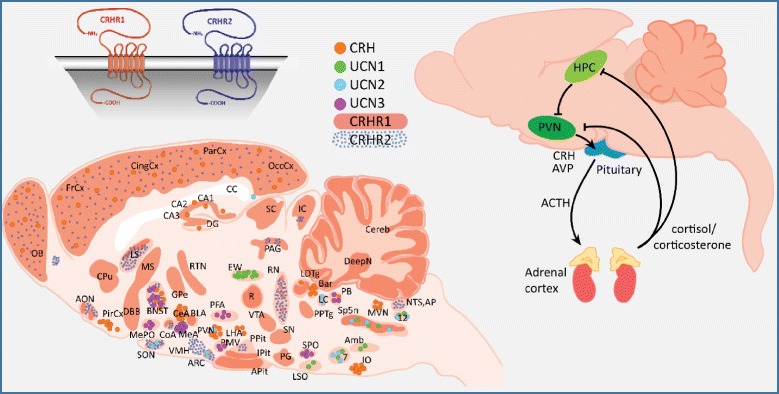
CRH-family expression and HPA axis. Adapted from [23, 67]

**Fig. 2:**
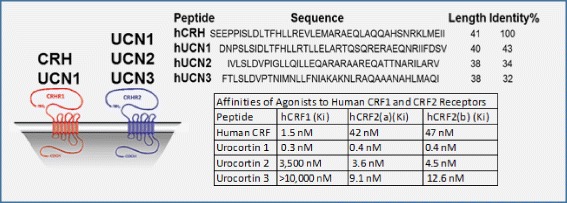
CRH-family peptides and receptor affinities [17]

In addition to the CRHR1 present in the anterior pituitary, the CRHR1 is also widely expressed throughout the brain (Fig. 1). Its signaling in these locations is thought to cause many of the psychologic effects of stress and is also implicated in learning and memory [20–22]. Indeed, CRHR1 is expressed on both glutamatergic and dopaminergic neurons and can have either anxiogenic or anxiolytic effects depending on the brain region [23]. It appears that stress, up to a certain threshold, is stimulating and induces rapid memory formation; above that threshold, stress becomes overwhelming and deleterious [23, 24]. CRH expressing neurons are distributed throughout the CNS. Of special relevance in AD is the expression of CRH in the cortex and hippocampal-amygdalar complex, areas which also express the CRHR1 receptor [15]. The role of the CRHR2 receptor is not yet fully understood; it has been implicated in the anorexic effects of stress, and theories as to its role vary from functioning as a counterbalance to CRHR1 stress signaling, to being a part of a different stress pathway that responds to inescapable and unpredictable stressors [16]. The CRHR2 is expressed in discrete areas of the brain, but is widely expressed in the periphery (Fig. 1) [25]. The urocortins, similarly to the CRHR2, are expressed discretely within the brain. Both CRHR1 and CRHR2 are G-Protein Coupled Receptors that signal primarily through the G_s_, G_q_, and MAPK/ERK pathways [15]. Activation of these pathways can lead to increased intracellular calcium levels [26], an effect that may be worthwhile to explore considering the hypothesized role of calcium dyshomeostasis in AD [27]. In terms of specificity towards CRHR1 and 2, i) CRH has specificity for the CRHR1, ii) urocortin 1 can activate both CRHR1 and CRHR2, and iii) urocortin 2 and 3 are unique in that they show specificity for CRHR2 and do not appear to have affinity for CRHR1 at physiologic concentrations [15, 28] (Fig. 2). Overall, CRH and the urocortins are proposed to act as neuromodulators within the CNS to orchestrate various components of the psychologic stress response [17].

### Cortisol and AD

In the 1990’s and early 2000’s, an appreciable amount of study focused on the role of cortisol and corticosteroids in AD; this was largely driven by several observations. One of the direct effects of psychologic stress is an increased level of circulating glucocorticoids, mainly cortisol in humans and corticosterone in mice [18]. Cushing’s syndrome, a state of excess glucocorticoids seen in patients with pituitary tumors or those who have received large amounts of exogenous steroids, is associated with atrophy of the hippocampus, a feature shared with both conditions of chronic stress and AD [29–31]. The hypothesized role that glucocorticoids play in AD has been reviewed elsewhere [31–33], but we will make note of the most critical data. AD patients have been shown to have mildly increased levels of cortisol when compared to controls (Table 2) [34, 35] and corticosteroids have been shown to be able to induce Aβ plaque load and hyperphosphorylated tau levels in APP overexpressing mouse models [36–39], effects that were able to be reduced with a glucocorticoid receptor (GR) antagonist [40].

**Table 2:** Relevant stress biomarker studies in AD

Biomarker studies	Description	Results	Comments
Bernardi et al. (2000) [42]	Serum levels of Cortisol and two neurosteroids, Allopregnanolone and dehydroepiandrosterone (DHEA), were compared in AD patients and controls	AD patients had significantly higher levels of cortisol and lower levels of Allopregnanolone compared to age matched controls	Clinical Dx of AD *N* = 12
Popp et al. (2015) [34]	Longitudinal prospective study examining serum and CSF levels of cortisol and Aβ in age matched controls, patients with mild cognitive impairment (MCI), and AD patients.	CSF cortisol levels are elevated in subjects with AD and MCI. Elevated CSF cortisol levels were associated with faster cognitive decline in MCI of the AD type.	Clinical Dx of AD
Ennis et al. (2016) [35]	Prospective trial measuring once yearly a 24 h urinary cortisol level, over an average interval of 10.56 years	Elevated urinary cortisol level was related with a 1.31 times increase in AD risk, predicting increased AD risk an average of 6 years before onset.	Clinical Dx of AD. Participants that went on to be grouped in the “Future AD” group were 10 years older on average than control group

The hippocampus is a key negative regulator of the HPA axis, with increased glucocorticoid levels causing increased GR signaling in the hippocampus leading to inhibition of CRH release from the hypothalamus [18]. One hypothesis for the increased levels of glucocorticoids found in AD is that hippocampal damage may cause a loss of negative feedback on hypothalamic CRH release. AD patients have also been found to have altered responses to a CRH stimulation test, with higher levels of cortisol than controls [41, 42], suggesting that AD patients may have a more complex alteration in HPA axis activity.

### CRH and AD

In the early 2000’s, investigation into the stress response and AD began to branch into the CRH signaling pathway. This occurred simultaneously with a large effort to target CRH signaling in various stress related disorders (Major Depression, PTSD, addiction). Since this time, a substantial amount of data implicating CRH signaling in both Aβ production and tau phosphorylation has been produced.

### Aβ

An increasing amount of evidence shows that stress and specifically CRH-CRHR1 signaling can increase Aβ peptide production. In primary neuronal cultures (PNC’s), CRH treatment was shown to increase levels of total secreted Aβ peptide, an effect that was partially blocked by the small molecule CRHR1 antagonists Antalarmin and R121919 [43]. Our group was able to replicate the CRH-induced increases in Aβ in both PNC’s and a CRHR1 overexpressing SH-SY5Y cell line, but we saw similar increases upon treatment with the peptide CRHR1 antagonist Astressin as were seen with CRH treatment [44]. Recent work showing that adrenergic GPCR’s are able to affect APP processing led to our hypothesis that the CRHR1 may also be able to directly regulate these processes [45]. An in vitro γ-secretase assay showed that CRH treatment can modulate γ-secretase activity towards increased Aβ42 production [44] and that upon CRH treatment, CRHR1 is internalized by a β-arrestin 2 mediated interaction, where it then colocalized with γ-secretase intracellularly. This fit well with previous data that has shown that β-arrestin regulates Aβ production via y-secretase [46]. Furthermore, we showed that the peptide CRHR1 antagonists Astressin and α-helical CRH were able to induce rapid internalization of cells overexpressing the CRHR1. This data led to the hypothesis that CRHR1 antagonists may modulate γ-secretase activity towards increased Aβ production through inducing internalization of the receptor. Evaluation of the G_s_ coupled pathway of CRHR1 has shown that pretreatment with the Protein Kinase A (PKA) inhibitor H-89 was able to significantly reduce Aβ40 and Aβ42 production in a CRH treated PNC model [43]. Through these investigations, a picture begins to form that includes three possible mechanisms for CRH induced Aβ increases.

1. **CRHR1 interactions with β-arrestin 2 and γ-secretase:** demonstrated by the data that CRHR1 antagonists do not stimulate the receptor, but do induce its internalization and increase Aβ40 and Aβ42 production in vitro [44].
2. **CRH directly modulating γ -secretase activity:** demonstrated by data that CRH treatment increases Aβ42 production in an in vitro γ–secretase assay [44].
3. **Classical CRHR1 G**_**s**_ **/G**_**q**_ **pathway signaling:** demonstrated by data that treatment with PKA inhibitor H-89 is able to reduce CRH induced increases in Aβ40 and Aβ42 in vitro [43].

Multiple stress paradigms have been able to induce increases in Aβ40 and Aβ42 levels and plaque load in the brains of wild type (WT) and mutant APP overexpressing transgenic mouse models (Table 3).

**Table 3:** Studies involving stress paradigms and Aβ production

Studies on Aβ	Model	Stress paradigm	Effect	Comments
Kang et al. (2007) [47]	Tg2576 [82]	3 months isolation	↑	
Dong et al. (2008) [48]	Tg2576	6 months isolation	↑	
Lee et al. (2009) [83]	Tg2576	2 h restraint daily for 16 days	↑	Measured ISF, soluble Aβ
Ray et al. (2011) [50]	Wistar rat	5 h restraint or bilateral UCN1 injection in amygdala	↑	
Huang et al. (2011) [84]	APP/PS1 [85]	4 months isolation	↑	
Rothman et al. (2012) [86]	3xTg AD [87]	Cage switched for 6 h/day for 2–3 days/week for 6 weeks	↑	
Dong et al. (2012) [49]	Tg2576/ CRH overexpressing [88]	CRH overexpressing transgenic	↑	
Rothman et al. (2013) [53]	3xTg AD	6 h sleep restriction for 6 weeks	-	Aβ and tau measures trending upward but non-significant
Dong et al. (2014) [43]	Tg2576	One week or 10 months of isolation	↑	
Baglietto-Vargas et al. (2015) [89]	3xTg AD	5 h of multimodal stress. Brief restraint, shaker plate with bright lights and noise	↑	
Park et al. (2015) [44]	C57BL/6 J	3 h restraint	↑	
Justice et al. *(*2015) [90]	APP/PS1	PTSD-like induction for two hours followed by a trigger	↑	

Studies in WT mice or rats observed levels of soluble Aβ42 and Aβ40 in brain homogenate, studies in transgenic mice included measurements of plaque load and insoluble Aβ unless noted otherwise

Studies investigating the mechanisms through which stress mediates these effects on Aβ have focused mainly on CRHR1 and GR signaling. While acute restraint stress was able to induce increases in total soluble Aβ levels in the brain interstitial fluid (ISF) of Tg2576 mice, these increases were not reproduced with administration of corticosterone to the mice. These increases *were* able to be induced by the administration of CRH and blocked by administration of the CRHR1 antagonist α-helical CRH [47]. These findings suggest that acute stress-induced increases in Aβ40 and Aβ42 in the brain may be due to a CRH-mediated effect, which agrees with previous data showing that corticosteroid-induced increases in brain Aβ40 and Aβ42 levels take place 48–72 h after administration [36]. In addition, it was found that chronic isolation stress caused increases in both GR and CRHR1 expression in the cortex and hippocampus, along with increased corticosterone levels and plaque burden in the Tg2576 mouse model [48]. In a double transgenic Tg2576 and CRH overexpressing mouse model, it was shown that overexpression of CRH was related with large increases in brain Aβ40 and Aβ42 levels along with increased plaque burden in the cortex [49].

While the role of the urocortins in stress-induced Aβ production has been largely unexplored, one study found that injection of urocortin 1 (an agonist for both CRHR1 and CRHR2) into the amygdala of male Wistar rats induced increases in amygdalar Aβ40 levels, while 5 h of acute restraint stress in the rats showed increases in both Aβ40 and Aβ42 [50]. A double transgenic Tg2576 and homozygous CRHR1 knockout mouse line (supplemented with corticosterone for health maintenance) showed highly significant decreases (>60%) in basal levels of Aβ40, Aβ42, and APP C-Terminal Fragment (α and β) levels in the brain, suggesting that a CRHR1-mediated mechanism contributes to an appreciable amount of Aβ processing [51].

### Tau

While there is a substantial amount of evidence that CRH signaling can affect Aβ production and Aβ plaque deposition, most data connecting the CRH signaling pathway with tau-related processes is shown through studies on tau phosphorylation and not aggregate deposition. The neurofibrillary tangles found in the AD brain are composed of hyperphosphorylated, cleaved, and conformationally altered tau protein [52]. Phosphorylation of tau at certain residues causes its dissociation from microtubules and enables aggregates to form [52].

Multiple stress paradigms have been able to increase tau phosphorylation in mice [53–60] (Table 4) and it was shown that a CRH KO mouse model did not show the stress induced increases in hyperphosphorylated tau that were seen in WT mice [57].

**Table 4:** Studies involving stress paradigms and tau phosphorylation/aggregation

Studies on Tau	Model	Stress paradigm	Effect	Comments
Rissman et al. (2007) [55]	C57BL/6 J, multiple CRHR knockouts	Acute restraint at multiple time points up to two hours. Also done in adrenalectomized mice.	↑	
Carroll et al. (2011) [54]	PS19 [91]	Variable stressor once per day for 4 weeks, or restraint six hours/day and isolation for one month	↑	Variable stress paradigm not consistent in elevating tau measures
Rissman et al. (2012) [59]	CRHR Knockouts	Half hour restraint one time or daily for 14 days	↑	
Filipcik et al. (2012) [57]	C57BL/6 J	30 min or 120 min restraint	↑	
Campbell et al. (2015) [51]	CRH overexpressing		↑	
Le et al. (2016) [58]	Primary Neuronal Culture from C57BL/6 J	Treatment with CRH	↑	

Studies utilizing WT mice observed tau phosphorylation at various phospho-epitopes. Study utilizing PS19 mice quantified inclusions

Furthermore, it was shown that stress induced tau phosphorylation could not be replicated by administration of corticosterone and that CRHR1 antagonist NBI27914 was able to decrease the effect of stress on tau phosphorylation [54] at certain phospho-epitopes. Mice that had undergone adrenalectomy also replicated the stress-induced increases in tau phosphorylation. These data provide several pieces of evidence pointing away from a glucocorticoid mediated mechanism and towards one mediated by CRH [55]. It was also shown that a CRHR1 knockout mouse line did not replicate these increases in stress induced tau phosphorylation, while their CRHR2 knockout counterparts actually had a hyperactive response to stress when compared to WT mice, lending evidence to the hypothesis that the CRHR2 may have a regulatory role over CRHR1 signaling [55]. A CRHR1 and CRHR2 double knockout mouse maintained the lack of response seen with the CRHR1 single knockout mouse [59]. Lastly, a CRH overexpressing mouse model showed increased tau phosphorylation at multiple sites when compared with controls [56]. Unfortunately, the vast majority of these studies on stress and tau pathology have used WT mice and only measure tau phosphorylation and not aggregate formation. While increased phosphorylation of tau frequently correlates with increased aggregation and neurodegeneration in models of tau pathology, further evaluation of the CRH signaling pathway in deposit-forming models of tau pathology is necessary. As transgenic models of tau aggregation are actually models of Frontotemporal Dementia (FTD), these studies could additionally link stress to other dementias or neurodegenerative processes.

### Interventions targeting the CRH signaling pathway

With evidence showing that both CRH and glucocorticoids can exacerbate AD pathology in the brains of transgenic mouse models, it follows that abnormalities in the HPA axis may play a role in the development and progression of AD. Targeting CRH could be the most efficacious single intervention within the HPA axis, as an intervention blocking the effects of CRH would decrease downstream glucocorticoid release, potentially blocking both CRH-mediated and glucocorticoid-mediated effects on Aβ production and tau phosphorylation.

Overactive CRH signaling has been implicated in anxiety, major depression, and inflammatory disorders and an effective intervention targeting the CRH signaling pathway is highly sought in these fields [16, 17, 61, 62]. Despite preclinical studies demonstrating their potential efficacy, for unknown reasons, the CRHR1 antagonists GSK511679 and Pexacerfont were not shown to be effective in clinical trials investigating their effects in alcoholism and generalized anxiety disorder [63]. While there has yet to be a clinical trial investigating an anti-CRH therapy in any neurodegenerative disease, there has been substantial effort to implement CRHR1 antagonists preclinically in mouse models of Aβ and tau pathologies (Table 5). CRHR1 antagonists have shown some efficacy in decreasing stress induced Aβ pathologies, with no effect in an acute stress paradigm [44], but alleviating effects in chronic stress paradigms [43] as well as in unstressed APP overexpressing mice [64]. Studies have utilized both peptide and small molecule CRHR1 antagonists (Astressin, Antalarmin, α-helical CRH, R121919, NBI27914) in the various aforementioned stress paradigms. One study found that even in unstressed mice, the CRHR1 antagonist R121919 was able to significantly decrease amyloid deposits in the brain. The effects of the CRHR1 inhibitors on tau showed either decreased tau inclusions in the cortex of PS19 tau mice [54] or decreased tau phosphorylation [43, 59], but, in contrast to the work done in various APP overexpressing mouse models, the investigation into stress and tau aggregation is limited to a single study.

**Table 5:** Studies testing CRHR1 antagonists in mouse models and in vitro models relevant to AD

CRHR1 antagonist	Studies	Dosage	Outcome	Comments
Antalarmin	Rissman et al. (2012) [59], Dong et al. (2014) [43], Park et al. (2015) [44],	20 mg/kg	Rissman (2012) - Able to block acute stress induced tau phosphorylation but not that induced by chronic stress [59]. Dong (2014) - Able to decrease Aβ levels and plaque load in a chronic stress paradigm [43]. Park (2015) - Unable to block rises in Aβ in response to acute stress [44].	Dong (2014) measured PBS soluble Aβ, which represents roughly 5% of total Aβ.
A-helical CRH	Park et al. (2015) [44]	5 & 10 μM	Unable to block CRH induced Aβ increases in vitro.	
Astressin	Park et al. (2015) [44]	5 & 10 μM	Unable to block CRH induced Aβ increases in vitro.	
NBI 30775/R121919	Rissman et al. (2012) [59], Campbell et al. (2015) [51], Dong et al. (2014) [43], Zhang et al. (2016) [64]	20 mg/kg. Zhang (2016) utilized osmotic mini pump	Campbell (2015) [51] – able to decrease AT8 and PHF-1 phosphorylation in CRH overexpressing mice but not at S262 and S422. Rissman (2012) [59] – Able to decrease stress induced AT8 and PHF-1 increases in phosphorylation	
NBI27914	Park et al. (2015) [44], Lee et al. (2009) [83], Carroll et al. (2011) [54]	10 mg/kg	Carroll (2011) [54] - NBI27914 rescued freezing and decreased AT8 phosphorylation and decreased neurodegeneration in PS1 tau transgenic mice. Lee (2009) [83] – NBI27914 reduced stress induced increases in Aβ levels and plaque load. Park (2015) [44] – NBI27914 increased secreted Aβ42/ Aβ40 ratio in vitro	

Although there has been some proof of efficacy for CRHR1 antagonists in decreasing AD pathology in mouse models, one should question the probability of their success in human trials, as these antagonists have a history of having promising effects in rodent models and then failing in the clinic [61, 62, 65, 66]. In addition, CRHR1 antagonists can induce the internalization of CRHR1 and cause increases in Aβ40 and Aβ42 in in vitro models of the human CRHR1, drawing some concern to their use as an AD prevention. We believe that the strength of the literature shows that the CRH signaling pathway is an interesting target that merits further investigation into possible therapeutic interventions, but we posit that direct targeting of CRH will provide stronger and more worthwhile effects than receptor-based interventions. Direct targeting of CRH could be accomplished through several methods including, but not limited to, CRISPR-CAS9, antisense oligonucleotides, or direct immunotargeting of the CRH peptide. Any investigator attempting to develop one of these methods can do so with the knowledge that the creation of a therapy able to effectively downregulate CRH signaling would have broad impact in many conditions of chronic stress and possibly AD.

### Impact of psychologic stress on other neurodegenerative diseases

In addition to AD, psychologic stress has been explored as a risk factor and exacerbating component in Parkinson’s Disease, Huntington’s Disease and vascular dementia. There are several comprehensive reviews available on the impact of psychologic stress and Major Depression on neurodegenerative disease [32, 67, 68], and it is likely that stress acts through unique mechanisms in each disease. Similarly to AD, increased psychologic stress has been associated epidemiologically with an increased risk of Parkinson’s Disease [69, 70]. Stress paradigms have been shown to exacerbate the phenotype of several mouse and rat models of Parkinson’s [71–76], yet there do not appear to be studies attempting to target stress pathways therapeutically within these models. Furthermore, early reports have shown that stress paradigms have a detrimental effect on the phenotype of the R6/1 mouse model of Huntington’s [77, 78]. Further investigation into the mechanisms of how stress signaling affects these proteinopathies is needed, and should also grow to include other neurodegenerative proteinopathies such as Amyotrophic Lateral Sclerosis (ALS) and FTD. Indeed, as mentioned earlier some of the work already done on tau mouse models may be applicable to FTD. Lastly, it is pertinent to mention that the risk of both stroke and vascular dementia have also been associated with increased psychologic stress levels [11, 79, 80]. As patients with all of the neurodegenerative conditions mentioned suffer from stressful neuropsychiatric comorbidities [67, 81], interventions targeting stress signaling pathways could have both preventative and therapeutic effects on these diseases.

## Conclusions

Considerable epidemiologic data suggests that chronic psychologic stress can be a risk factor in AD, or at least dementia. Studies in animal and cell models provide biological underpinnings to support this association and identify CRH as the logical therapeutic target. Based on current data, successful antagonism of CRH may result in beneficial effects on both Aβ amyloid and tau pathology, an effect that would be predicted to have a major impact on AD. More speculatively, such therapies could improve the harmful negative behavioral symptoms that accompany chronic stress. Indeed, normal stress-signaling is not always deleterious and plays a role in learning and memory. Empirical studies will need to be carried out in order to determine if one can target CRH directly in a manner that has beneficial effects and avoids side effects due to under-stimulation of the pathway. Given the need for novel targets in AD, we believe that additional preclinical studies that explore the potential impact of targeting CRH could pave the way for increased interest in this pathway as a target in AD.

## Abbreviations

ACTH: Adrenocorticotropic Hormone
AD: Alzheimer’s Disease
ALS: Amyotrophic Lateral Sclerosis
Aβ: Amyloid β
CRH: Corticotropin-releasing hormone
CRHR1: Corticotropin-Releasing Hormone Receptor 1
CRHR2: Corticotropin-Releasing Hormone Receptor 2
FTD: Frontotemporal Dementia
GR: Glucocorticoid Receptor
HPA: Hypothalamic-Pituitary-Adrenal
NFT: Neurofibrillary tangles
PKA: Protein Kinase A
PTSD: Post-traumatic Stress Disorder
PVN: Paraventricular nuclei
UCN: Urocortin
WT: Wild type
